# Analysis of events from sudden isolated dysarthria to diagnosis of myasthenic crisis: myasthenia gravis mimicking acute lacunar stroke—a case report

**DOI:** 10.1186/s13256-024-04617-w

**Published:** 2024-07-04

**Authors:** Simona Petkovic Miletic, Sheikh Ruksana Binte Ahmed

**Affiliations:** 1 Acute Medicine Unit, Epsom General Hospital, Dorking Rd, Epsom, KT18 7EG UK; 2 Epsom General Hospital, Dorking Rd, Epsom, KT18 7EG UK

**Keywords:** Myasthenia gravis, Stroke mimics, Lacunar stroke, Isolated dysarthria, Case report

## Abstract

**Background:**

Myasthenic crisis (MC) is a life-threatening complication of myasthenia gravis (MG), necessitating ventilation. Achieving a safe and timely diagnosis of myasthenic crisis with atypical, isolated presentation is a considerable challenge particularly in elderly patients, where myasthenia gravis can present with isolated dysarthria in rare instances, giving a clinical impression of lacunar stroke.

**Case presentation:**

We present a compelling case of a 73-year-old Caucasian female presenting with abrupt onset of isolated dysarthria. Despite initial treatment for a presumed lacunar stroke, subsequent evaluations led to her diagnosis of a myasthenic crisis. Within 72 h of admission, the patient developed dysphagia and shortness of breath, requiring supplemental oxygen. The case highlights the sequential progression of events from the atypical presentation of isolated dysarthria and its course to the management of a myasthenic crisis.

**Conclusion:**

Our reported case focuses on the discussion of myasthenia that mimicked a lacunar stroke and was finally diagnosed at a critical time of medical crisis. This case highlights the imperative notion that isolated dysarthria in elderly individuals warrants vigilant monitoring for possible myasthenia gravis, given the low incidence of lacunar stroke presenting with only dysarthria.

## Introduction

Myasthenia gravis (MG) is an acquired immune-mediated neuromuscular transmission disorder characterized by reduced functional acetylcholine receptors (AChR) on the postsynaptic membrane of the neuromuscular junction. This gives rise to the fluctuating fatigue of skeletal muscles, frequently including symptoms of extrinsic ocular muscle impairment or bulbar involvement, resulting in a neurological emergency termed myasthenic crisis [1]. While MG traditionally exhibits a bimodal distribution affecting younger women and older men, it can also develop in other age groups. Our presented report primarily focuses on tackling the diagnostic complexities associated with atypical presentations of myasthenic crisis, specifically within the elderly population. Notably, a study showed that the proportion of newly diagnosed MG patients over 70 years old is as high as 59% within the study cohort [2]. MG is under-diagnosed in elderly individuals, at times confused with cerebrovascular disease. This diagnostic challenge is exemplified in a case series of myasthenia patients presented with isolated sudden dysarthria, leading to the misinterpretation of symptoms as those of a stroke [3]. The risk of misinterpretation of MG as cerebrovascular disease accentuates the imperative for increased awareness and consideration of MG as a viable differential diagnosis in the presentation of sudden onset isolated dysarthria.

The primary aim of our distinctive case report is to highlight the significance of promptly identifying isolated sudden dysarthria as a potential atypical presentation of myasthenic crisis (MC) in older individuals. This instance of MG, resembling lacunar stroke, emphasizes the necessity for thorough assessment in elderly patients to avert unfavourable outcomes. The absence of preceding MG symptoms in such scenarios accentuates the diagnostic complexities, prompting clinicians to contemplate MG in elderly patients presenting solely with dysarthria and to implement comprehensive clinical approaches.

## Case presentation

### Patient information

A 73-year-old Caucasian female presented to A&E with a stroke call, reporting the sudden onset slurring of speech. Additionally, she recounted a brief episode of blurred vision the day before the admission, which had spontaneously resolved. Her past medical history included hypertension, Type 2 diabetes mellitus, hypercholesterolemia, an uneventful recovery from a right total hip replacement performed five weeks prior to presentation, and no known adverse obstetrics history with two daughters. Prior admission, she was independent of activities of daily living, living alone. She was noted to have no smoking or alcohol excess history on initially obtained history in stroke call. While the patient’s slurred speech had been noted during a telephone call prior to admission, the exact timing of symptom onset remains uncertain as she had not spoken to anyone for 14 h before this and hadn’t noticed any changes in her speech herself. Patient was on atorvastatin 10 mg oral once a day, omeprazole 20 mg once a day and Candesartan 16 mg once a day, before admission to hospital.

### Clinical findings

During the stroke assessment on admission, the patient's vital signs were recorded as follows: blood pressure 147/87 mmHg, pulse 88 beats/min, temperature 36.8 degrees Celsius, and oxygen saturation of 96% on room air. On systemic examination, she had clear lungs on auscultation as well as normal cardiovascular and gastrointestinal examination. Both the stroke and medical teams reviewed the patient upon admission and found her to be alert and responsive, with a Glasgow Coma Scale of 15/15. On neurological examination, she was able to follow commands, had normal horizontal extraocular movements, intact visual fields, symmetrical facial nerve distribution, 5/5 muscle power in all four limbs, and intact sensory distribution. However, she exhibited dysarthria, resulting in a National Institutes of Health Stroke Scale (NIHSS) score of one, attributed to mild to moderate slurring of speech. The patient also passed the swallow test according to the stroke protocol upon admission. An urgent CT head revealed no acute haemorrhage but indicated moderate small vessel disease.

### Timeline

On Day 1 of admission, post urgent CT head, the patient was started on 300-mg aspirin and 80-mg atorvastatin. She was admitted to the Acute Medical Unit for further management, including an inpatient MRI brain and carotid Doppler on the next working day. Over the following 48 h (Days 1 and 2) from admission, the patient was provisionally treated as a suspected small lacunar infarct with persistent stable mild dysarthria. Blood tests for stroke workup, including thyroid function test, HbA1c, and inflammatory markers were unremarkable. Routine chest X ray was also noted to be normal.

During the medical ward round on day 3, the patient reported feeling exhausted with her ongoing dysarthria. However, in this instance, she reported a new feeling of a lump in her throat, prompting a referral to the speech and language assessment team. After review, they recommended switching her medications to a liquid formulation, with a follow-up plan the next day. On the same afternoon, the acute medicine team was informed that the patient could not tolerate an MRI brain due to shortness of breath while lying flat. After consulting with the stroke team, a repeat CT head was arranged while the patient continued to be managed for a presumed lacunar stroke.

**Fig. 1:**
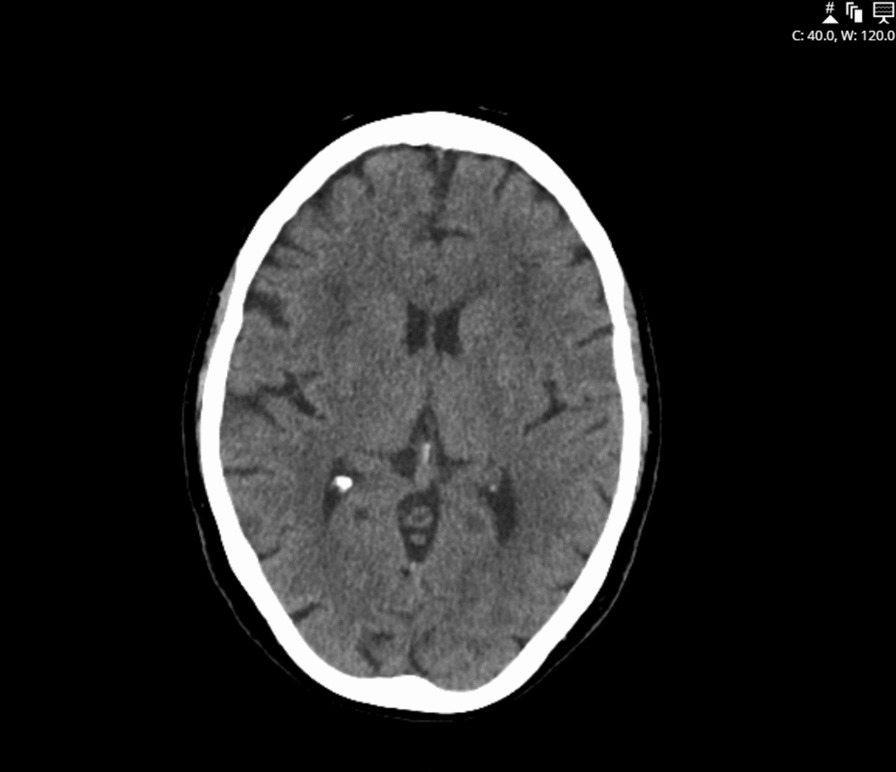
Computerised tomography head on stroke call showing no acute bleed, but moderate small vessel disease

Following the repeat CT head on day 4 morning, the patient developed new shortness of breath required 1L oxygen. On thorough examination during the morning ward rounds, she had new mild bilateral ptosis, a prominent nasal voice, and general fatigue with normal muscle power. Her repeat CT head from Day 4 was similar to the previous report on admission, showing no acute intracranial abnormality (Fig. 1). At this point, considering her ongoing dysarthria, new-onset ptosis, and new oxygen requirement the patient was given a new working diagnosis of a possible myasthenia gravis, by the acute medicine team. Thus, an immediate sample for AChR antibody testing was sent, and urgent neurology consultation was sought.

### Diagnostic assessment

The diagnosis of MG in this case was confirmed with a comprehensive neurology review on the afternoon of Day 4, following the diagnostic suspicion of MG by the acute medicine physician on the ward round. During the review, the patient exhibited bulbar dysarthria with pronounced fatigable ptosis on the left, particularly noticeable during upward gaze, and mild fatigable proximal upper limb weakness. Additionally, the patient demonstrated heightened shortness of breath during a conversation with the inability to lie flat in the bed, indicative of respiratory muscle involvement of MC. Recognizing these critical symptoms of respiratory support requirement in MC, the medical team promptly initiated treatment for the MC and urgently referred her to the intensive care unit (ITU) for close monitoring, with the guidance of the neurologist. Given the urgency, the clinical diagnosis of MC was made based on examination and symptom evolution, bypassing the wait for diagnostic investigations before taking immediate action for MC management.

**Fig. 2:**
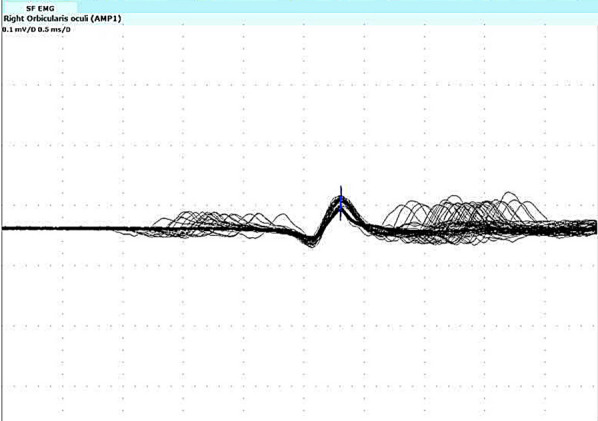
Single fibre electromyography study on right orbicularis oculi showed 8 paired potentials, diagnostic for myasthenia gravis

In the ITU, a nerve conduction study (NCS) was conducted, revealing abnormal findings in the repetitive nerve stimulation study (Fig. 2, Table 1). The jitter study performed further supported the diagnosis of severe MG. Simultaneously, a CT chest scan was conducted, which showed no evidence of thymoma, a common association with MG [4]. Furthermore, the patient’s acetylcholine receptor (AChR) antibody test returned positive, providing additional confirmation of the MG diagnosis. This multifaceted approach, including critical clinical evaluation to initiate crisis management, further neurophysiological studies, imaging, and serological testing, contributed to a comprehensive and conclusive diagnosis of severe MG in the context of an MC.

**Table 1:** Repetitive nerve stimulation study showed both facial nerves and nasalis muscle showed a decrement of 28% on both sides

Repetitive nerve stimulation study					
Muscle	Event	Time	Amp Decr mV	Amp Decr %	Amp Decr %
Right ADM – Adb dig minimi (hand)	Run 1	11:04:40	5.8	− 0.15	− 3.3
	Run 2	11:05:01	5.7	− 1.17	− 6
	Run 3	11:05:13	4.8	− 3.9	− 7.5
	Run 4	11:06:47	5.8	− 1.25	− 3.5
Left nasalis	Run 1	11:21:35	0.23	− 28.5	− 34.9
	Run 2	11:21:48	0.18	− 22	− 17.5
	Run 3	11:22:00	0.17	− 36.1	− 37.9
	Run 4	11:22:59 (Act + 00:24)	0.22	− 43.8	− 65.2
Right Nasalis	Run 1	10:57:09	0.57	− 29.6	− 12.6
	Run 2	10:58:20	0.6	− 28.2	− 20.5
	Run 3	10:59:11	0.57	− 12	− 6

All muscle fibres showed raised jitter with prominent blocking

### Therapeutic intervention

The specific management actions in this case highlight the need for swift intervention in MC. Upon diagnosing the MC, the patient’s treatment plan for lacunar stroke continued concurrently, driven by the unavailability of an MRI. In response to the clinical diagnosis of MC, immediate pharmacological measures were initiated with efficient hospital pharmacy and nursing team, including the administration of intravenous Immunoglobulin at 2 g/kg, divided over five days, as per neurology advise. Recognizing the risk of thromboembolism with Immunoglobulin therapy, subcutaneous low molecular-weight heparin 5000 units once per day was concurrently administered. The treatment regimen included pyridostigmine at 30 mg four times daily and a prednisolone schedule starting at 5 mg for three days, progressively increasing to 50 mg/day.

Given that a significant majority, ranging from two-thirds to 90%, of MC patients require intubation and mechanical ventilation [5, 6], the patient was admitted under ITU from the point of crisis diagnosis. This decision was intended to secure the airway within the specialized environment of an intensive care unit, after urgently initiating immunomodulatory and immunosuppressant therapy in the acute medicine setting. Taking into account the preference for elective intubation in myasthenic patients facing impending respiratory failure as opposed to emergent intubation [6], the patient was subsequently electively intubated to facilitate optimal management.

During the patient's stay in the intensive care unit (ITU), she completed the 5-day course of Intravenous Immunoglobulin and continued on an escalated dose of oral prednisolone at 50 mg/day, along with pyridostigmine at 60 mg four times daily. However, due to the high dose of steroid therapy, the patient developed steroid-induced diabetes and was consequently commenced on oral metformin at 500 mg three times a day, in addition to gliclazide tablet at 80 mg once daily in the morning.

Following endotracheal intubation by 3rd day of ITU admission, elevated inflammatory markers were observed (Table 2), prompting initiation of broad-spectrum antibiotics IV Piperacillin and Tazobactam at 4.5gm three times daily. Within 48 h, the sepsis screen revealed staphylococcus pneumonitis (Table 3) in the endotracheal tube suction sample culture, leading to a switch to IV Ceftriaxone at 2 g once a day for 9 days, until normalization of inflammatory markers. After a week in the intensive care unit, the patient was successfully extubated upon improvement, following one failed extubation trial due to interim airway swelling. On completion of the intravenous antibiotics course, she was discharged on co-trimoxazole at 480 mg once daily to prevent opportunistic infections during steroid therapy.

**Table 2:** Investigations with trend chart from admission to hospital till follow ups

Timescale	On admission to hospital	3rd day of admission to ITU	On discharge	Follow up
*Investigation*				
White cell count (per microliter)	8.7	14.1	14.1	14.7
Haemoglobin (gram/deciliter)	117	103	115	116
Mean cell volume (femoliters)	89	87	86	89
Platelet count (per microliter)	289	219	292	183
Neutrophil (per microliter)	5.4	12.3	12.5	13.5
Lymphocytes (per microliter)	2.6	1.1	1.4	1.6
ESR (mm/hour)	73	–	–	–
C reactive protein (milligram / liter)	14.9	166.6	3.8	–
Sodium (millimole / liter)	139	130	137	135
Potassium (millimole / liter)	3.8	4.1	4.4	4.1
Urea (millimole / liter)	4.1	9.5	7.7	10.4
Creatinine (micromole / liter)	68	68	60	68
eGFR (milliliters per minute per 1.73 m^2^)	77	77	87	76
Bilirubin (micromole / liter)	6	6	5	7
Albumin (gram / liter)	34	23	28	21
Globulin (gram / liter)	31	40	35	-
ALT (units / liter)	9	7	35	20
Free T4 (picomoles/ liter)	15.9	–	–	–
TSH (mIU / liter)	1.61	–	–	–
Urine analysis	White cell > 35 * 10^6^ / L, Red cell < 10 * 10^6^ /L, Casts: none seen			

ITU Intensive care unit

**Table 3:** Microbiology investigations with result chart during Intensive care stay

Microbiology and serology investigations	Results
Influenza A & B	Negative
Coxiella Burnetti	Negative
Mycoplasma	Negative
Legionella pneumophillia Urine Antigen	Not detected
Pneumococcal	Not detected
Hepatitis C Ab, Hep B Surface antigen	Negative
HIV Ag/Ab	Negative
Urine MC&S	No growth
Sputum MC&S	No growth
Endotracheal tube suction sample	Staphylococcus Pneumoniae Sensitive to Penicillin, Erythromycin

Upon discharge, she was also commenced on lansoprazole at 30 mg once daily, cholecalciferol at 400 units/calcium carbonate at 1.5 gm twice daily, Candesartan tablet at 16 mg once daily, and loperamide at 2 mg once daily as needed with follow-ups with the neurology team and diabetic specialist team.

### Follow up and outcomes

The patient attended her first neurology clinic follow-up within four weeks of discharge, showing overall improvement in many myasthenic symptoms. However, she continued to experience occasional coughing with different food consistencies, and her speech would become more nasal towards the end of the day. To address the worsening symptoms towards the end of the day, the prednisolone dose was increased to 60 mg once daily, and Pyridostigmine was adjusted to 60 mg for the first two doses of the day and 90 mg for the end of the day in two doses. Additionally, she was switched from loperamide to Propantheline, taking 15 mg three times daily as needed to counteract potential faecal urgency from the increased pyridostigmine dosage.

During subsequent neurology clinic follow-ups, her pyridostigmine doses were adjusted to 60 mg four times a day, as tolerated. With the increase in steroid dose, she underwent an urgent review by the diabetic specialist team for uncontrolled hyperglycaemia, resulting in an increase in metformin to 1 g in the morning and 500 mg twice daily for the rest of the day and gliclazide to 180 mg in the morning and 60 mg in the evening. Her oral hypoglycaemics were eventually escalated to Humulin I insulin, starting from 12 units once daily with a further up titration plan for better glycaemic control, by diabetes specialist team clinic.

The patient experienced a setback with remission of MG within four months of discharge due to a COVID infection causing shortness of breath. Upon review in the neurology clinic, shortness of breath was attributed to the viral chest infection in the community rather than MG, and she was referred to respiratory physicians for outpatient review and spirometry.

At the six-month follow-up since the diagnosis of MG, she was started to begin a pyridostigmine weaning regimen, gradually reducing the dosage by 30 mg every two weeks until side effects such as diarrhoea, abdominal cramps, and sweating abated. During this evaluation, there was no signs of ophthalmoplegia or eyelid ptosis, no apparent voice fatigue, normal neck strength with palatal movement, and a healthy cough reflex. Consequently, the prednisolone dose was scaled back to 50 mg once daily, with a plan for ongoing clinic follow-ups aimed at further dose reduction over the next six-month reviews. Despite the improvements of MG symptoms, persistent side effects from pyridostigmine and the continued steroid tapering plan prompted the initiation of Azathioprine at 50 mg twice daily, with a strategy for dose adjustment and subsequent follow-ups as required.

## Discussion and conclusion

This case study outlines an elderly woman initially diagnosed with suspected lacunar stroke, whose evolving symptoms of ptosis and respiratory distress culminated in a diagnosis of myasthenia gravis (MG) during a myasthenic crisis (MC). It emphasizes the critical importance of considering MG as a potential diagnosis in elderly patients with isolated dysarthria, underlining the complex diagnostic challenges. Vigilant reassessment, especially when symptoms differ from typical patterns, is essential for timely and precise diagnosis and management.

As for most neurological illnesses, a meticulous history and physical examination are vital to the diagnosis of MG. There has been a notable surge in the number of individuals diagnosed with MG, surpassing a twofold increase over the past two decades, as reported in 2021. This upward trajectory is primarily linked to a heightened occurrence of MG among the elderly, possibly stemming from advancements in diagnostic techniques, improved treatment alternatives, and the overall extension of life expectancy [7]. Despite MG being a rare disease in Australia, it has an annual crude prevalence rate of 117.1 per 1 million residents, with peak rates observed between the ages of 74 and 84 in both genders, declining thereafter [8]. Due to its broad spectrum of manifestations diagnosis can be difficult with such diverse manifestations, and clinical suspicion is paramount. Ptosis is the leading indicator of MG. With age-related changes in eyelid appearance making it more noticeable in younger individuals, diagnosing it in older individuals may be challenging due to overlapping visual symptoms with age-related ocular conditions such as macular degeneration or cataracts [9]. In a Southern China report, 82.0% of MG-diagnosed individuals exhibited only ocular symptoms at onset, emphasizing the prevalence of ocular manifestations of MG [10]. Additionally, older adults’ fatigue-associated attribution of ocular symptoms further complicates the diagnostic process. The exacerbation of MG symptoms with muscle use and improvement with rest can also conceal the cardinal proximal muscle weakness prevalent in this population.

Approximately 80% of patients with MG have Acetylcholine receptor (AChR) antibodies, which cause AChR loss and postsynaptic membrane damage via complement activation [11]. While the association of AChR Antibody and muscle-specific kinase Antibody (MuSK-Ab) with MG is well-recognized, recent studies have identified a subset of double-seropositive MG patients. This subgroup experiences more pronounced bulbar palsy dysfunction and a higher incidence of myasthenic crises [12]. Although, this antibody profiles aid in long-term treatment planning, electrodiagnostic testing, including nerve conduction studies and repetitive nerve stimulation, remains the gold standard for diagnosing neuromuscular disorders [13] In repetitive nerve stimulation, a decrement of the muscle action potential and the muscle twitch was found in 72% and 49% of a study case, respectively; the diagnostic yield is proportional to the severity of MG [14]. Single-fiber electromyography (SFEMG) demonstrates high sensitivity as investigation modality of MG (88–92%) [15] but limited with specificity, as increased jitter in SFEMG for MG depends on the proportion of neuropathy or myopathy [16]. While the Edrophonium (Tensilon) test and repetitive nerve stimulation tests are less sensitive and specific, a positive assay for AChR antibodies is highly specific for MG, though it may have limitations in cases of purely ocular-muscle weakness MG [17].

MG treatment aims to induce remission and enable patients to resume their baseline functionality [18]. Currently, four primary treatment modalities are commonly used for MG treatment: anticholinesterase agents, immunosuppression, short-term immunotherapies such as plasma exchange and intravenous immune globulin, and review for surgical thymectomy. Addressing MC in this case study report, which occurs in approximately 20% of newly presenting MG patients [19, 20], the two primary pharmacological therapies are intravenous immune globulin (ivIG) and plasma exchange. Both treatments are equally effective in managing MG crises or significant MG relapses [21]. Upon prompt clinical recognition of the crisis, patients need to be started on treatment IV Immunoglobulin or plasma exchange, in a close high dependency unit setting. Due to MG patients having unique circumstances and co-morbidities, the immunosuppressive therapy prescribed needs to be customized individually, with the neurologist’s support.

Global epidemiological studies confirm a rising incidence of late-onset MG among individuals aged 65 and older, particularly those with multiple existing co-morbidities [22, 23]. Among these co-morbidities in the elderly age demographic, stroke is one of the most prevalent ones. About 75 percent of strokes occur in individuals aged 65 or older, with the risk doubling every decade after the age of 55 [24]. Given the high rate of stroke cases, particularly in patients with multiple co-morbidities across the mean age of 68.2 years (standard deviation: ± 15.6) [25], stroke tends to be the first provisional diagnosis for symptoms of dysarthria or limb weakness within this age group. However, MG is an example of a rare stroke mimic that presents a challenge to diagnosis within the same demographic. Two case studies are also reported to show myasthenia presenting with posterior circulation stroke symptoms [26]. Another retrospective review of twenty-one cases of elderly patients initially treated as an acute cerebrovascular event for stroke-like symptoms that were subsequently diagnosed with new-onset MG. Among this group, slurred speech (8 among 21 cases, 38.1%) was the most common symptom that resulted in misdiagnosis of stroke in the first place [9]. It is crucial to remember that although MG commonly occurs in younger populations, it can also manifest later in life. A hospital-based study showed that approximately 13 to 20% of their studied MG patients developed their first symptoms during the seventh decade of life or beyond [27]. The high prevalence of previously unrecognized positive AChR antibodies in those ≥ 75 years old suggests that myasthenia gravis may be substantially underdiagnosed in older people [28].

While MG is a rare condition, lacunar stroke with isolated dysarthria is also unusual. Isolated dysarthria is present in only 0.4% of patients with lacunar infarction [29]. and associated with dysarthria-clumsy hand syndrome in 2 percent [30], emphasizing the need for vigilance. The limited occurrence, at 6.8% in non-classic lacunar strokes [31], topped with the even rarer manifestation of subtype stroke cases with isolated dysarthria, emphasizes the imperative for physicians to contemplate the prospect of MG with such cases. The diagnostic challenge between MG and lacunar stroke with isolated dysarthria persists, given that CT scans rarely identify lacunar ischemic insults within the first 24 h due to their small size, unless there is a haemorrhagic component. In the acute and subacute settings, MRI emerges as a superior imaging modality to detect lacunar infarctions, with diffusion-weighted imaging (DWI) offering the highest diagnostic accuracy [32]. Until an MRI head confirms lacunar stroke, sudden onset isolated dysarthria with a normal CT head in the elderly population should prompt consideration of MG as a crucial and viable differential diagnosis. In conclusion, while stroke is a primary consideration in older patients due to their increased risk of cerebrovascular events, the growing diagnosis of MG in the elderly population warrants attention.

Our case of MG mimicking a lacunar stroke, diagnosed during a myasthenic crisis, illustrates the importance of a vigilant evaluation of elderly patients with similar symptoms to prevent an unfavourable outcome. It is unclear if the undiagnosed MG in this patient rapidly escalated to the level of myasthenic crisis with acute respiratory failure due to the high dose of atorvastatin started for ischaemic stroke treatment. Our patient clarified having no previous symptoms of myasthenia, such as fatiguability or ptosis, apart from the short-duration diplopia before admission. Additionally, the potential influence of hip surgery performed 4 weeks prior to admission on the onset of symptoms remains uncertain, as the recovery from the surgery was uneventful. Given that ischaemic stroke has a high index of suspicion in elderly patients, a stroke mimicking myasthenia gravis diagnosis is often delayed in this elderly patient group. We propose that clinicians encountering isolated dysarthria in an elderly patient should also consider MG, in addition to ischaemic stroke. A clinical approach with careful history and consideration of possible stroke mimics, followed by confirmatory studies, such as NCS, is crucial. This could ensure patient safety and prevent potentially life-threatening complications, which are treatable if recognized early on.

## Data Availability

All data underlying the results are available as part of the article and no additional source data are required.

## Abbreviations

MG: Myasthenia gravis
A&E: Accident and emergency
CT: Computerised tomography
MRI: Magnetic resonance imaging
HbA1c: Haemoglogin A1c
AChR: Acetylcholine receptor
NCS: Nerve conduction study
SFEMG: Single-fiber electromyography
ITU: Intensive care unit
